# Prevalence of Behavioural and Psychological Symptoms of Dementia and Its Association with Quality of Life in Dementia and Care Giver Burden–A Hospital-Based Study in Sri Lanka

**DOI:** 10.1192/bjo.2026.11098

**Published:** 2026-06-30

**Authors:** Amodha Medagedara, Kapila Ranasinghe

**Affiliations:** 1 ELFT, Luton, United Kingdom; 2 Faculty of Medicine Ragama, University of Kelaniya, Ragama, Sri Lanka; 3 NIMH, Angoda, Sri Lanka

## Abstract

**Aims:**

Behavioural and psychological symptoms of dementia (BPSD),though an integral feature of dementia leading to greater mortality and morbidity, remains understudied in low- and middle-income countries like Sri Lanka. Thus this study is performed to determine prevalence of BPSD and its association with quality of life (QoL) and care giver burden among patients attending Deeghayu clinic at the National Institute of Mental Health (NIMH), Angoda, the largest psychogeriatric facility in Sri Lanka.

**Methods:**

A descriptive cross-sectional analysis was carried out at the first clinic visit over a period of one year from 2024 to 2025. Prevalence was as certained with Neuro Psychiatry Inventory (NPI).

**Results:**

Total of 72 patients were diagnosed with dementia, 27.8% were females with a mean age of 76.9 years (SD±11.1). Mean duration of untreated symptoms by the time of diagnosis was 2.07 years (SD±1.54). Prevalence was 61.1% in Alzheimer’s, 4.2% in vascular, 30.5% in mixed (Alzheimer’s with vascular), 1.4% each in Parkinson’s, Lewy body and fronto-temporal dementia. Frequency of severities were 43% mild, 41.7% moderate and 15.3% severe. Severity of dementia was positively correlated with NPI score (Pearson Correlation 0.09). Prevalence of BPSD was 95.8%. 33.3% had delusions, 47.2% hallucinations, 54.2% agitation/aggression, 47.2% depression, 25% anxiety, 15.3% elation, 47.2% apathy, 19.4% disinhibition, 44.4% irritability, 65.3% wandering behaviour, 75% sleep disorder and 47.2% loss of appetite. 15.3% of the patients were initiated on donepezil, 5.6% on memantine, 4.2% on both. 13.9% were initiated on quetiapine alone for management of BPSD and 61.1% on quetiapine along with one or two cognitive enhancers. 51.4% had mild to severe care giver burden according to ZaritCare Giver Burden Scalewhich was positively correlated with NPI score (Pearson Correlation 0.29). All domains of QoL assessed via World Health Association QoL in both care givers and patients were negatively correlated with NPI score except for patients’ social and environmental QoL.

**Conclusion:**

Majority of the patients had BPSD at the time of diagnosing dementia associated with statistically significant heightened care giver burden and diminished QoL. This raises a concern that BPSD had led to accessing services rather than early cognitive and functional decline potentially indicating delayed diagnosis. Thus, this study underscores the need to enhance awareness and screening strategies to promote early detection of dementia in under-resourced settings such as Sri Lanka.

